# Unique Properties of Surface-Functionalized Nanoparticles for Bio-Application: Functionalization Mechanisms and Importance in Application

**DOI:** 10.3390/nano12081333

**Published:** 2022-04-13

**Authors:** Faheem Ahmad, Mounir M. Salem-Bekhit, Faryad Khan, Sultan Alshehri, Amir Khan, Mohammed M. Ghoneim, Hui-Fen Wu, Ehab I. Taha, Ibrahim Elbagory

**Affiliations:** 1 Department of Botany, Aligarh Muslim University, Aligarh 202002, India; khanfaryadamu@gmail.com (F.K.); amirsz2504@gmail.com (A.K.); 2 Department of Pharmaceutics, College of Pharmacy, King Saud University, Riyadh 11451, Saudi Arabia; salshehri1@ksu.edu.sa (S.A.); eelbadawi@ksu.edu.sa (E.I.T.); 3 Department of Microbiology and Immunology, Faculty of Pharmacy, Al-Azhar University, Cairo 11884, Egypt; 4 Department of Pharmacy Practice, College of Pharmacy, AlMaarefa University, Ad Diriyah 13713, Saudi Arabia; mghoneim@mcst.edu.sa; 5 Department of Chemistry, National Sun Yat-Sen University, Kaohsiung, 70, Lien-Hai Road, Kaohsiung 80424, Taiwan; hwu@faculty.nsysu.edu.tw; 6 School of Pharmacy, College of Pharmacy, Kaohsiung Medical University, Kaohsiung 807, Taiwan; 7 College of Pharmacy, Northern Border University, Arar 1321, Saudi Arabia; ibrahim.elbagory@nbu.edu.sa

**Keywords:** nanomaterial, bio-fabrication, magnetic nanoparticles, nanomedicine, nanocarrier

## Abstract

This review tries to summarize the purpose of steadily developing surface-functionalized nanoparticles for various bio-applications and represents a fascinating and rapidly growing field of research. Due to their unique properties—such as novel optical, biodegradable, low-toxicity, biocompatibility, size, and highly catalytic features—these materials are considered superior, and it is thus vital to study these systems in a realistic and meaningful way. However, rapid aggregation, oxidation, and other problems are encountered with functionalized nanoparticles, inhibiting their subsequent utilization. Adequate surface modification of nanoparticles with organic and inorganic compounds results in improved physicochemical properties which can overcome these barriers. This review investigates and discusses the iron oxide nanoparticles, gold nanoparticles, platinum nanoparticles, silver nanoparticles, and silica-coated nanoparticles and how their unique properties after fabrication allow for their potential use in a wide range of bio-applications such as nano-based imaging, gene delivery, drug loading, and immunoassays. The different groups of nanoparticles and the advantages of surface functionalization and their applications are highlighted here. In recent years, surface-functionalized nanoparticles have become important materials for a broad range of bio-applications.

## 1. Introduction

Nanotechnology is concerned with nanostructures that are smaller than 100 nanometers in at least one dimension and that can be altered at the atomic or molecular level. It is an interdisciplinary research field that includes chemistry, biology, engineering, and medicine, and it has great potential in early symptom diagnosis and treatment in clinical and environmental research [1,2]. The word ‘nano’ means a one-billionth scale of physical magnitude, and one nanometer is a one-billionth of a meter or 0.000000001 m [3]. In recent years, nanoparticles (NPs), mainly metal NPs, have expanded in biological sciences to be used for diagnosis and therapeutics purposes. That extension in use and applications of NPs can be explained by their distinctive small size, as well as their enormous surface-area-to-volume ratio, high responsiveness to living tissues, stability at elevated temperatures, and cellular transportation [4].

Newly synthesized nanoparticles encounter barriers, i.e., fast agglomeration and oxidation that hinder them from expanding their uses in broad fields. This problem can be addressed by surface functionalization, stated as, “the incorporation of a chemical functional group to the surface of nanocomposites allows for surface modification that permits for self-organization, compatibility, and the possibility for a variety of applications” [5]. Organic and inorganic functional groups both impart an important role in the surface modifications of atoms of desired metals or metal oxides. This approach not only prevents nanoparticles from oxidizing and agglomerating, but also allows for enhanced functionalization [6]. Simple organic molecules/groups render enough potential defense to nanoparticles against agglomeration by linking with the surfaces of other nanoparticles, molecules, or solids [7]. In this way, NPs can get closer to a biological entity of interest. They can potentially be coated with biological molecules and enable them to bind with or adhere to the surface of a biological unit, allowing for a controlled method of identifying or targeting it [8].

NPs should typically be compatible with both the focused biological systems and properties required for water solubility. Despite the fact that there are evolved strategies for coating NPs in order to ensure water solubility and preferred features, perfect surface coating should permit for particular criteria such as preventing unwanted NP accumulation during long-term storage, retaining good water solubility, preserving NP features, and guaranteeing biocompatibility prior to NP communication with targeted disciplines [9]. In biology and medicine, successful applications of NPs are sometimes required for entry into living cells, which means that NPs need to cross a significant barrier, i.e., the cell membrane, a nm-scale lipid bilayer containing embedded or peripherally bound proteins [10]. NPs can be inserted into living cells through many well-known methods: (a) non-specific endocytosis, in which NPs commonly end up in endocytic partitions; (b) straightforward microinjection of nanoliter of NP scattering, which is a time-consuming process and only applicable to a limited number of cells; (c) electroporation, which utilizes charges to force NPs across the membrane; and (d) mediated/targeted ingestion based on NP surface functionalization via biological interactions or promoters [10]. Nanotechnology has the power to play a critical role in a variety of customized drug applications. In recent years, there has been a rise in the number of nanotechnology strategies developed to overcome the drawbacks of targeted therapies [11,12], enhanced medical visualization and diagnosis of diseases [13,14], pest management [15], improvement of crop production [16], theranostics operations incorporating diagnostic and therapeutic innovations [17,18], and development of biosensors and biosensing gadgets [14,17]. Nanoparticles (NPs) are widely applied as nanomedicine and nanocarriers for drug delivery [18,19].

In this review, we first concisely introduce the nanoparticles and the elements affecting why surface modification of magnetic nanoparticles is essentially required, followed by the mechanism of surface functionalization with various organic and inorganic materials. Thereafter, we briefly highlight some important nanoparticles—such as iron oxide, gold, silver, platinum, and silica-coated—with their potential applications in diverse areas.

## 2. Nanoparticles and Surface Functionalization

**Figure 1:**
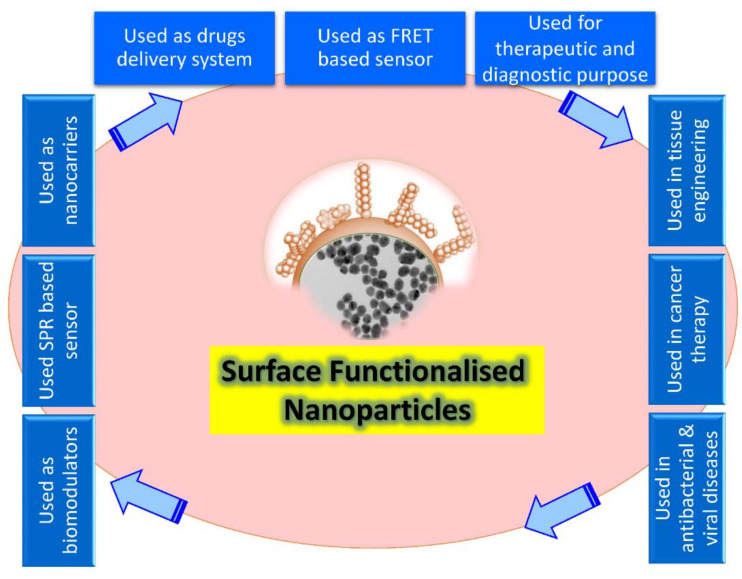
Schematic illustration showing the broad array of bio-applications of surface-functionalized nanoparticles.

Recently, the level of NPs has been considered the most advanced, whether in scientific research or in commercial applications. A nanoparticle size is significant in its physiological and biochemical properties and diverse formats such as fullerenes, metal NPs, ceramic NPs, and polymeric NPs. Their nanoscale size and high surface area give NPs unique physical and chemical properties. NPs’ shape and structure affect their reactivity and toughness, their size affects other properties as well [20]. Surface recognition by NPs provides a possible tool to control the cellular and extracellular processes for various biological applications such as enzymatic inhibition, delivery, sensing, and transcription regulation (Figure 1). Depending on the core material, NP cores can be altered from more minor to larger sizes, giving an appropriate platform for the NP interactions with proteins and other biomolecules [21].

Fabrication could be defined as the creation of complicated biological goods utilizing living tissue, molecules, extracellular arrays, and designed biomaterials based on these variables. However, this limits fabricated goods to living organs and tissues [22], but still suggests that natural resources have to include living or bio-inspired content, constituent procedures should be biology-based, and byproducts must be complex living cells [23]. On the contrary, according to a more comprehensive definition, bio fabrication includes a diverse set of physical, chemical, biological, and/or technological aspects with a variety of applications in biological sciences, including environmental and bioprocess monitoring, food quality control, agriculture, bioterrorism, and medical biosensor systems (Figure 1).

### 2.1. Interactions Involved in Surface Functionalization

Surface functionalization of nanoparticles applies to the use of covalent and non-covalent bonds—such as hydrogen bonds, electrostatic force, and the van der Waals interactions—to integrate diverse organic and inorganic molecules at the nanoscale [24]. Typically, multiple linker molecules are used to form covalent bonds between ligands and the surfaces of NPs. Pagels et al. [25] reported that PEG can be produced with specified functional groups at the terminals and employed as heterobifunctional linkers to execute a variety of functionalization activities. Non-covalent interactions have the advantage of simplicity and not influencing the structure of the molecules utilized or their interactions with target biological materials. However, the different factors—such as pH and ionic strength—can easily influence non-covalent changes [26]. Different types of nanostructures have distinct chemical characteristics and functional groups coated on their surfaces that can be employed in the early stages of surface functionalization. In general, the very first step of surface modification involves the use of homo- or hetero-bifunctional cross linkers with the goal of adding an organic functional group (R–NH_2_, RCOOH) and many more for binding biological molecules. There are three major aspects behind the surface functionalization of nanoparticles, including: firstly, the stabilization of nanoparticles against agglomeration and oxidation processes [6,7]; secondly, to make them compatible with a subsequent phase, i.e., when suitable ligands are linked to metal particles, they can be made water soluble [27]; thirdly, to help them organize themselves [5]. The surface alteration enhances the mechanical characteristics of the nanocomposite by avoiding homogeneity and compatibility issues between the two phases [28].

### 2.2. General Protocols and Material Required for Surface Functionalization

**Figure 2:**
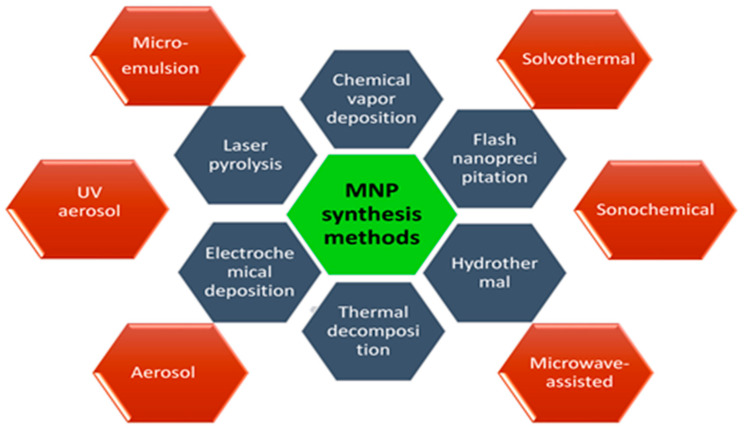
General methods of surface functionalization of magnetic nanoparticles (MNPs).

As we know the size, shape, morphology, and dispersibility of nanoparticles can all have a significant impact on their biological applications. As a result, scientists are concentrating their efforts on surface modification of nanoparticles using various approaches to regulate their size, shape, and morphology while retaining variable and beneficial features. A number of synthesis approaches have been explored and reported to prepare the magnetic nanostructures such as chemical vapor deposition [29], flash nanoprecipitation [30], hydrothermal [31], thermal decomposition [32], electrochemical deposition [33], laser pyrolysis [34], microemulsion [35], solvothermal methods [36], sonochemical methods [37], the microwave-assisted method [38], aerosol pyrolysis [39], and UV aerosol synthesis [40] (Figure 2).

## 3. Mechanism of Surface Functionalization of NPs

Bioorganic and bioinorganic chemistry also provides a basis to join biotechnology with the advanced materials science. For example, bioorganic model systems give tools for probing the mechanisms of biological principles and developing chemical methods to manage natural components. Organic ligands and inorganic nanoparticle surface interaction facilitates coupling the biomolecular recognition systems in order to create novel materials (Figure 2). The formed multilayered laminate properties may be controlled in several ways, involving changing the adsorbing substances in the dipping solutions. The driving force for substance adsorption also depends on the nature of both the surface and adsorbing substance. Therefore, organic and inorganic compounds both significantly play a key role in the stabilization of newly synthesized nanoparticles through surface functionalization.

### 3.1. Surface Modification with Organic Molecules

Because of their simple structure, organic molecular agents can only be used in one way (ligand exchange or ligand adsorption) and monodentate ligands are commonly employed in ligand exchanges due to their ease of synthesis, simple structure, and other benefits. Recently, the organic materials have included citrates, phosphates, amines, thiols and various polymers (chitosan, dextran, PEG, polyvinyl alcohol (PVA), poly(lactic-co-glycolic acid) (PLGA), alginate, ployacrylic acid, pollunan, etc.). Polymer-coated IONPs have received much attention in the past few years because of their variety of uses in numerous research fields, including nanomedicine. Two typical methods for making polymer coated IONPs are in situ and post-annealing coating [41]. Ali et al. [42] used an emulsion polymerization process to create Janus-like MNPs by modifying poly(methylmethacrylate-acrylic acid-divinylbenzene). The synthesized Janus-like MNPs ranged in size from 200 to 250 nm in diameter. The main advantage of MNPs with small molecular coatings is that large hydrodynamic sizes (>50 nm) may be addressed. MNPs can be successfully fabricated with specific groups—such as –COOH, –NH_2_, –OH, and –SH—which can be further manipulated with various bioactive molecule connections. Dheyab et al. recently used a quick and simple one-step co-participation technique to successfully construct highly magnetic and 19 nm-sized citric-acid-functionalized MNPs. The results showed a magnetic saturation of 54.8 emug^−1^ [43]. Chitosan is a nontoxic, alkaline, biocompatible, biodegradable, and hydrophilic polymer. To make a ferrofluid, Lee et al. [44] produced spherical MNPs and implanted them in chitosan. They discovered in vitro that MNP-chitosan nanoparticles enhanced MR image contrast significantly as compared to ferrofluids.

### 3.2. Surface Modification with Inorganic Molecules

Inorganic molecules consisting of silica, metals, and metal oxides [34,45,46,47,48] are in use. MNP surfaces modified with organic molecules have strong biocompatibility and biodegradability, in addition to the basic magnetic properties. For example, various organic compounds—including oleic acid, 1-octadecene, 1-tetradecene, and oleylamine—are typically added to the chemical reaction as stabilizers to obtain narrow size distribution iron oxide nanoparticles (IONPs). The well-known Stöber method is the most popular method for producing MNP@SiO_2_ nanomaterials [49]. In situ, SiO_2_ is produced through hydrolysis and condensation of a sol–gel precursor. The Stöber method is a versatile technique that can be utilized in both aqueous and organic conditions.

Surface coating of IONPs with metallic elements as an inorganic compound is also employed to improve their stability, biocompatibility, and disperstivity. The noble metal gold is the most widely utilized for surface coating. Generally, there are two ways to get a gold-shell coating on the surface of magnetic IONPs: direct and indirect [50]. When gold is covered with a strong sulfur conjugation, it delivers tremendous benefits. The chemical inertness of gold allows gold-coated MNPs to be perfectly stable. Wang et al. [51] used gold precursors to reduce iron oxide nanoparticles of various sizes as seeds to synthesize gold-coated iron oxide nanoparticles.

### 3.3. Stabilization of Nanoparticles

**Figure 3:**
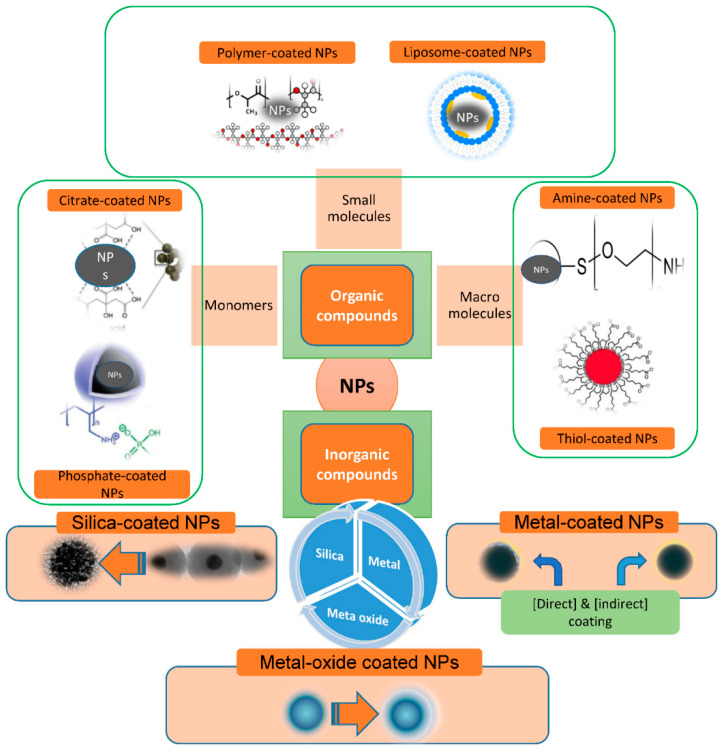
Scheme illustration for materials commonly used for nanoparticle (NP) functionalization.

The stabilizing slows the nucleation activity and influences the adsorption of modifiers on nuclei and developing nanocrystals, potentially inhibiting IONP growth and favoring the development of micro IONPs. When compared to naked MNPs, nanocomposites functionalized with inorganic substances can have significantly improved antioxidant activities. The inorganic molecules—including silica, metals, metal oxides, nonmetals, and sulfides—are mainly exposed to atom surfaces. Surface modification of MNPs with silica (SiO_2_), gold (Au), and silver (Ag) is prevalent, producing core–shell patterns and ensuring nanoparticle integrity in solution. They also aid in the binding of numerous biological substances and medicines to the MNPs’ surface via appropriate functional moieties [52,53,54,55] (Figure 3).

The surface functionalization of nanoparticles can be achieved through two modes: (i) In situ modification, a single step method which involves the synthesis and surface functionalization simultaneously; (ii) Post-synthesis modification, a two-step procedure in which synthesis of NPs is followed by the modification of surface. The coating procedure is determined by the coating compounds’ properties as well as the desired application. Generally, ligand addition, ligand exchange, or encapsulation are used to modify the surface of NPs [56,57,58].

### 3.4. Ligand Addition

**Figure 4:**
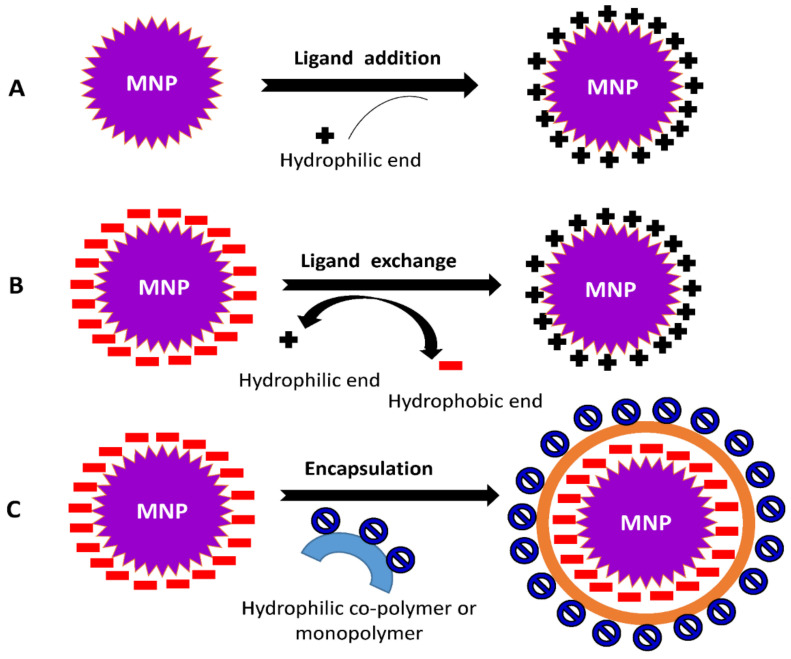
Common mechanisms involved in surface modification of NPs (**A**) ligand addition, (**B**) ligand exchange, and (**C**) encapsulation.

It is the process of adding a ligand to the exterior surface of produced NPs without removing any previously attached ligands (Figure 4A). The following strategies are required for ligand addition to NPs’ surface viz., (1) The ligand used is prepared with no capping agent; (2) Indirect addition of ligand, the formation of an inorganic layer on the surface of NPs followed by the direct adsorption of a ligand via ionic or other non-specific interactions; (3) Using the ‘hydrophobic attractive interaction’ to easily integrate hydrophobic molecules into NP’s hydrocarbon shell capped by desired ligands; and (4) A covalent connection is formed between the present ligand and the approaching ligand.

### 3.5. Ligand Exchange

This process involves changing a hydrophobic ligand into a hydrophilic one. These ligands are typically made up of hydrophilic and binding groups. The binding groups can attach to the NPs’ surface, exposing the hydrophilic groups to the external environment and enabling them to completely dissolve in the aqueous solution (Figure 4B).

### 3.6. Encapsulation

During the encapsulation procedure, NPs covered with hydrophobic ligands are coated with amphiphilic compounds. The hydrophobic component of materials is intercalated with the initial ligand on the surface of the MNPs, resulting in the hydrophilic portion facing the solution (Figure 4C). The head groups within the hydrophilic region of the substances allow MNPs to become water-compatible.

## 4. A Brief Note on the Unique Properties and Importance of Some Nanoparticles

### 4.1. Iron Oxide Nanoparticles (IONPs)

Iron oxide is the comprehensively investigated compound in biomedical techniques as it is characterized by high biocompatibility in relation to other magnetic resources, based on both the oxides and pure metal. Even though there are various forms of iron oxides that occur in the environment and they may be synthesized in the lab, only maghemite (γ-Fe_2_O_3_) and magnetite (Fe_3_O_4_) can meet the standards for biological applications. These parameters involve chemical stability, high magnetic moments, and low toxicity. In addition, there a simple and economical procedures to prepare these materials [45]. The efficacy of nanoparticles in diagnostic or therapeutic approaches is based upon a number of criteria, including the quantity of atomic order in iron oxide, crystallinity, and dispersity in regards to NP size and shape [59]. Furthermore, iron oxide NPs are nonporous. This helps to prevent delay caused by diffusion, so equilibrium is reached quickly and a quick response is possible. The drawback here is their self-condensation response, which results in multiple layer structure or accumulative deposition on surface layer. Phosphates, on the other hand, do not go through self-condensation interactions. These agents form a stable Fe–O–P structure by combining with surface-OH groups [7].

The inherent aggregation behavior of iron oxide NPs happens due to a high surfac- area-to-volume ratio and the attraction forces among magnetite, an important and a limiting component that diminishes intrinsic super paramagnetic properties [60], and activates opsonization procedure [61]. Hence, reducing aggregation is essential to engineering iron oxide NPs’ surface in order to prevent the particles’ aggregation in biological solution to achieve more effective stabilization [62]. The used approaches are grafting or coating to organic compounds, such as silica, metal or non-metal elementary content, metal oxide, or metal sulphide. Basically, defending shells could be used to normalize magnetic oxide NPs and improve NPs’ effectiveness as aptamers in sensing and (bio)chemical laboratory tests [63]. Silica is frequently used as a coating material over the surface of NPs as it is chemically inactive and encourages NP dispersion.

Use of nanoscale materials in combination with biomolecules has resulted in development of novel breed of hybrid modified electrodes to improve charge transfer as well as bioactivity retention [64]. Applications of iron oxide nanoparticles in various fields are summarized in Table 1.

### 4.2. Gold Nanoparticles (AuNPs)

The capability of gold NPs (AuNPs) to interact with cells and to enter them has helped the researchers to attach different biological macromolecules and compounds to gold. Because they tend to respond and clump with other NPs, gold NPs are available in various sizes and shapes [20]. AuNPs are typically made up of a thin gold shell encasing a dielectric core (an insulating material such as silica), which is referred to as nano-shells or AuNPs (typically spherical) with a size range of 0.8 to 250 nm. They are also distinguished by high absorption coefficients [114]. The optical properties of AuNPs are determined by their size and shape. The optical resonance of gold may be altered and moved as far as the mid-infrared region by changing the width of the core and outer shell of AuNPs [115]. Functionalization of nanoparticles is necessary for their stability, functionality, and biocompatibility. The main goal in functionalization is to preserve properties of both the AuNPs and the bound biological molecule. Surface-functionalization of AuNPs is critical in biological devices, diseased areas to interact selectively with cells, or biological molecules [116].

In general, the functionalization of AuNPs can be achieved by either using chemical functional groups or biological molecules. AuNPs with diameters just under 10 nm exhibit a variety of physiochemical and thermodynamic applications due to their increased surface-area-to-volume ratio. Ligands have thiol groups which bind covalently to Au atoms during the reduction of the HAuCl_4_ and assemble into an outer layer on the Au crystals [117]. For NPs with a 2 nm gold core, the covalently attached ligands are tightly packed and organized into structures similar to lattices in crystals. The choice of ligands and their organization will bear their biological properties and NP interaction with macromolecules in solutions and on target cells. Because of their long-term stabilization; ease in controlling size distribution; and good compatibility with biomolecules such as antibodies, antigens, proteins, DNA, and RNA, AuNPs have been commonly used in numerous immunoassay techniques in recent years [118]. Gold nanoparticles (AuNPs) are primarily suitable for advancement of quick tests. Photothermal AuNPs are being used in medical applications such as cancer treatment [119]. The surface of AuNPs could be handled to improve their stability and specificity, as well as to decrease accumulation. Functionalized AuNPs are being used for gene transfection and silencing, focused drug or gene delivery, intracellular recognition, bioimaging, cancer care, and as biosensors [11,120]. Some of the gold nanoparticle applications in various fields are summarized in Table 1.

### 4.3. Platinum Nanoparticles (PtNPs)

Platinum nanoparticles (PtNPs) are extremely important due to its exceptional catalytic properties, that is influenced by ligands on their outer side. PtNPs can have a variety of surface functional groups that are hydrophilic, lipophilic, and chemically reactive [121]. Some platinum compounds are utilized as anticancer drugs because they are highly effective. This feature is related to DNA replication inhibition by adding PtNPs to DNA strands [122]. On the other hand, Hikosaka et al. [123] showed that PtNPs have a nicotinamide adenine dinucleotide (NADH) ubiquinone oxidoreductase-like activity. This finding suggests that PtNPs could be applied to ameliorate oxidative stress conditions defined by a lowered mitochondrial complex I [124]. Due to their affinity for DNA strands, PtNPs can be more effective as carriers for carrying drugs into bacteria. It is also a crucial element of fuel cells, where platinum acts as the very efficient electrocatalyst for oxygen reduction reaction (ORR) and fuel (which includes hydrogen, methanol, ethanol, and formic acid) oxidation reaction [125]. PtNPs catalytic activities mainly depend on their sizes, shapes, and structures [126].

Nowadays, there are various valid approaches to assembling nano-building materials, which include the use of preformed building blocks to construct NPs with high-ordered architecture. The methods of making well-organized NPs are categorized into biological and non-biological assemblies. These are utilized to produce complex hybrid nanomaterials containing unique biological or microbial compounds such as antigens and antibodies [127], glucose oxidase [128], DNA [129], and bacteria [130]. Ahmad et al. [131] reported a rapid detection of bacteria using antibody immunoglobulin G (h-IgG) to fabricate the platinum nanoparticle. The aggregation of NPs, particularly platinum, stimulated by immunoreactions provides a unique immunoassay process that uses light scattering identification to reach great sensitivity [132]. The study explained a potentially fast and effective immunoassay that used antibody-platinum NP adducts as a message particle [132]. This technology has many benefits: (a) easy to execute, (b) tiny quantity of reagent is being used, (c) provides a faster response, and (d) provides an effective and low-cost identification. The applications of platinum nanoparticles in various fields are summarized in Table 1.

### 4.4. Silver Nanoparticles (AgNPs)

A distinctive emergent silver-based material has been shown to offer a lasting antimicrobial advantage that can offer environmental control of pathogenic bacteria on treated surfaces. Silver NPs (AgNPs) are handy labels. They have huge optical cross-sections with excellent photostabilities [133]. They are also responsive to multimodal imaging including optical, electron, and X-ray. Silver has been explained in literature as a bulk material that is efficient against a wide range of pathogens [134]. Silver NPs have been examined and found to be highly attractive for use in several applications such as wound dressings, coatings for medical devices, and textile fabrics impregnation [55]. In spite of these benefits, it is challenging to prepare antibody functionalized AgNPs of a size that can generates sufficient signal for high temporal resolution optical imaging (20–40 nm) and remains stable in physiological buffers. Under these situations, screening the stabilizing charge of the NPs may lead to aggregation, and AgNPs can undergo oxidative corrosion [135]. Some improvement was achieved in synthesizing stable silver–DNA conjugates [136]. The direct method to biofunctionalized noble metal NPs is by using electrostatic attraction to have a non-covalent attachment of antibodies to the metal surface. The surface charge of AuNP complexes implies conformation of biomolecules on NPs and influences particle–biomolecule interactions, in addition to ensuring the stability of nanoconjugates [137]. Thiolation by organic thiol compounds is also a method of metal-surface functionalization. Under moderate circumstances, thiols generate stable metal–sulfur bonds using surface atoms in noble metals. Other thiol compounds immobilized on silver are 3-mercaptopropionic acid [138], 2-mercaptoethanol, 2-aminoethanethiol, and 2-mercaptoethanesulfonic acid sodium salt [139]. Functionalization of AgNP surfaces highly affects their characteristics. The optical properties of silver nanoparticles were altered after they were functionalized with alkanethiols. For each carbon atom in the alkane chain, the maximum of the local surface plasmon resonance spectra shifts linearly to the red by 3 nm. In contrast, only 60,000 alkanethiol molecules per nanoparticle cause spectral large shifts of up to 40 nm. Some of the silver nanoparticle applications in various fields are summarized in Table 1.

### 4.5. Silica-Coated Nanoparticle

Utilizing thin silica coating techniques, a variety of water-soluble, functionalized NPs can be synthesized [140]. Thin silica coating in the toluene stage was conducted utilizing triethoxysilane or trihydroxysilane, which takes the benefit of avoiding silica polymerization thus restricting the texture of silica shell, preventing particle–particle cross-linking, and generating narrower water-soluble NPs [20]. The thin silica coating was highly reproducible that can be used in a variety of hydrophobic NPs, including Au, Ag, Fe_3_O_4_, and quantum dots (QDs). The silanized granules that resulted were monodispersed, with high water solubility and colloidal consistency. PEG silane-coated NPs have recently been developed as contrast agents for magnetic resonance imaging (MRI) visualization of tumors [141]. A recently published article reported the functionalized thin silica-coated NPs with aptamers and antibodies and used them in biological applications [142]. Koole et al. [143] outlined an evolutionary approach for coating silica particles with a closely packed monolayer of lipids without the use of coupling agents in two steps. Step one involved integrating strongly monodisperse silica particles with a single thread QD in their center and making diethylene triamine penta-acetic acid (DTPA) bistearyl amide (DSA) in hydrophobic lipid coating [143]. They were made target-specific by multiple Rvβ_3_-integrin specific RGD conjugation (arginine glycine–aspartic acid) peptides to enable their recognition with both fluorescence techniques and MRI. Using imaging technology, it was discovered that lipid coating improved bio-applicability and pharmacokinetics [143]. A fluorescent contrast material was used to functionalize silica-coated granules in a sequential manner. The drug image sensitizer palladium–porphyrin payload has been used for therapeutic treatment, and biomolecular ligands c(RGDyK) peptides on outermost exterior were used to target cancer cells’ Rv-3 integrins [53]. A membrane which consists of bis-sorbylphosphatidylcholine, has been used to enhance both the coating environmental and chemical stabilities [144]. This system decreased nonspecific interactions and allowed the functionalization of the particles. A novel delivery system termed a ‘nano-shuttle’ was described with a nano scale PEGylated-phospholipid coating and a 13-(chlorodimethylsilylmethyl) heptacosane-derived mesoporous silica NP [145]. Some of the silica nanoparticle applications in various fields are summarized in Table 1.

## 5. Toxicity of Surface-Functionalized Nanoparticles

Because of the rising development of nanomaterials and their commercial applications, toxicity concerns are unavoidable. NPs are hazardous because of their wide surface area and chemical responsiveness, resulting in the production of reactive oxygen species that can penetrate cells and tissues. Natural mechanisms produced NPs are hazardous to humans facing exposure. Humans are exposed to NPs as they are produced by natural processes [146]. The toxicity of NMs is dependent on a number of parameters, such as dose and exposure time, aggregation and concentration, particle size, particle shape, surface area, crystal structure, surface functionalization, and pre-exposure [9,146,147]. The tiny size of NPs allows active compounds species to pass through living organisms boundaries such the skin, lungs, body tissues, and other organs. As a result, depending on their composition, NPs might cause irreversible oxidative stress, organelle damage, asthma, and cancer [9]. Inhaled NPs can easily enter the bloodstream and other parts of the human body, such as the liver, heart, and blood cells. It is important to note that the toxicity of NPs is determined by their source. Many of them appear to be harmless, while others appear to have beneficial effects on human health [148]. Free particles have been shown to be more harmful than fixed or linked MNPs, while magnetic nanoparticles have been reported to be more dangerous than their bulk form [58]. Bacteria-mediated NP production has been discovered to be extremely effective in nanomedicine usage because it reduces the risk of cellular toxicity [149].

Free NP movement is unrestricted, making it easier for them to disseminate across the environment and pose a serious health issue to humans. In contrast, careful handling of fixed NPs, wherein the nanomaterial components are connected to a large object, poses no health hazard. In biomedical applications, coated MNPs have several advantages over bare MNPs, particularly lower cytotoxicity, improved cytocompatibility, and enhanced bio-conjugation. The presence of reactive elements on the surface of MNP’s core and the shell’s composition delivers biocompatibility and bio-conjugation capabilities [58,150]. Another effective method in the functionalization of NPs is to combine amino acids and peptides, improving nanoparticle-based delivery systems’ specificity and efficacy. NPs functionalized with amino acids such as lysine, polylysine, and glycine bind DNA more effectively for gene delivery without causing toxicity. The principal ammonium groups of these amino acids aided in their ability to bind to cationic organizations on DNA [151]. Dextran-coated IONPs are recognized as potential prospects for biomedical applications due to their biosafety, bioactivity, biocompatibility, and minimal cytotoxicity [47].

## 6. Application of Surface-Functionalized NPs in Biological Sciences

### 6.1. Nano-Based Imaging

The bioimaging applications of nanotechnology for basic research are enhanced by the pursuit of NPs as drug-delivery devices and clinical diagnostics. This made it easier to get the attention of investors and translational medicine grants. The use of NPs in imaging applications continues to progress rapidly, both in the advancement of current agents and in the development of new ones. The method’s analytical properties demonstrated the importance of moment fluorescence imaging techniques in reducing interference of cellular auto fluorescence and specimen background fluorescence [152]. Although NPs play an essential role in medical strategies, their role in diagnostic imaging is still deemed inadequate. They have been approved by the FDA as therapeutic drugs for a variety of diseases, including cardiovascular, autoimmune, and chronic kidney disease [153]. The current worldwide economy for visualizing imaging methods is USD 5 billion, whilst the market for oncologic therapy drugs in the United States is, by itself, approximately USD 100 billion [154]. Iron oxide nanoparticles—such as Resovist, Feridex, and Gastro MaRK—are being used to improve MR pictures [155].

In contrast to conducting surgeries whether pre-operatively or intraoperatively in patients, the preoperative and intraoperative imaging techniques proved their capabilities to be used in reducing the residual tumor cells. These NPs would just provide image processing contrast, permitting surgeons to distinguish tumor margins [156]. The section that follows explains how NPs can be used in combination with different imaging modalities such as MRI, ultrasound, photoacoustic imaging, CT, and fluorescence imaging. The appearance of luminescence has been efficiently altered by integrating numerous dyes into NPs, which could be useful for multiplex imaging. Because of high spatial resolution, polymer stabilized magnetic NPs are being used for magnetic resonance imaging (MRI), tracking cell migration, and monitoring cell differentiation in vivo status [157]. Conjugating the iron magnetic NPs to antibodies which target the proteins expressed on the surface of human cancer cells may improve the accuracy of MRI to detect early-stage cancer [158]. Carbon or polymeric NPs labeled with fluorine-18 deoxyglucose were investigated in pre-clinical models to enhance both the diagnosis and detection rates of the tumors by using positron emission tomography [159].

The continued improvements of the nano-based difference means will help to image tumors with an extraordinary resolution. This will improve our understanding of disease progression and the determination of tumor location. Positron emission tomography (PET), single-photon-emission computed tomography (SPECT), and magnetic resonance imaging (MRI) were the first diagnostic methods to create images with molecular clarity. All such technologies investigate the ability to collect data on cellular anatomy, physiology, and metabolism. Therefore, more contrast agents and imaging modalities are being invented regularly to increase treatment. NPs have also been imaged with the goal of developing new drugs and improving the resection methods to offer quantifiable outcomes about treatment efficiency as evident from the study conducted by the European Society of Radiology [160]. The biological importance of nanoparticles based on imaging is summarized in Table 1.

### 6.2. Gene Delivery

In biology, nucleic acid delivery has now been thoroughly investigated and analyzed, and lipid-based gene delivery has already gained popularity, an important methodology for neuroscientists and biologists. Currently, nanotechnology-based delivery systems are utilized for gene therapy. NP surfaces could be functionalized with numerous substituents due to their surface-to-volume ratio, allowing them to be gene therapeutic agents messengers [161]. Nanotech based gene delivery for gene therapy is a significant and massive step forward into gene editing, which will alter our capacity to cure many incurable diseases. Thus, every gene therapy is created using comprehensive insights into the causes of a patient’s disease [161]. Since gene therapy became a viable option for precision medicine, the development of a system capable of targeted delivery to cells or tissues and delivery of medicinal genetic information became a top priority [162]. Targeted gene delivery enable the NPs to be used in a variety of applications, including cancer treatment [163]. Yet another useful feature for nano-base gene delivery is in genetically based immunization [164]. Utilizing molecules as nano-platforms with applicable antigenic moieties is an appealing substitute for conventional inoculations. Incorporation of antigens in nanoparticles can be attained by one of two methods; entrapment (physical encapsulation) or complexation (covalent functionalization) [165].

NPs were shown in research findings to safeguard existing antigen configuration from proteolytic cleavage and to improve antigen transmission to antigen-presenting cells (APCs) [166]. NPs can be injected subcutaneously and intramuscularly, and they have been supplied via mucosal sites (oral and intranasal). They are capable of penetrating capillaries and also mucosal surfaces [167]. ‘Gene therapy’ refers to the use of nucleic acids to cure and govern illnesses. One such form of therapy employs viral and non-viral vectors to transmit exogenous gene into somatic cells in order to cure faulty genes or perform other biochemical processes [161].

According to Han et al. [168], AuNPs were functionalized to cationic quaternary ammonium groups before being electrostatically bonded to plasmid DNA. The discharge of DNA from customized Au NPs after glutathione therapies also was noted. The target gene was apoB, and the effectiveness of RNA expression silencing was first studied in vitro utilizing mouse liver cells (FL83B). The in vivo silencing effectiveness was greatest at 1 mg per kg (an acceptable therapeutic dose) but did not increase beyond that density. According to result of this research, utilizing both cationically charged dendrimer and chemically treated siRNA successfully quiets oligonucleotide. A concept design of this type will enable the development of well-defined RNAi systems capable of successfully suppressing gene expression in a manageable and non-toxic manner. Surface-modified silica NPs were also used to deliver or identify nucleic acids [169]. Biological importance of nanoparticles as gene delivery are summarized in Table 1.

### 6.3. Drug Loading

A factor that delays clinical translation of nanomedicine is drug loading. Insufficient drug loading with uncontrolled drug release is one of the hardest challenges to overcome. Most nanomedicines have low drug loading. The clinical translation of nanomedicines is challenging because of high production costs, production scale-up problems with reproducible properties, and the possibility of toxicity. Additionally, very high particle concentration is needed to attain a therapeutic drug window. However, viscous solutions with elevated NP densities cause injection comorbidities [170]. As a result, drug loading boosting is critical to reducing these adverse effects [120]. On either side, with rising drug-loading NPs, only a tiny quantity of NPs are needed to achieve the desired therapeutic standard, which reduces the possibility of negative impacts from over-dosed substances and lowers the cost of nanomedicine [171]. These advantages become really advantageous for those drugs that are well accepted, allowing doses to be substantially enhanced. Drug loading is critical in the development of drug-loaded NPs. Multiple studies define drug loading and encapsulation efficacy, making it difficult to directly compare distinct NP systems. A most promising use of NPs is in drug delivery. They should ideally have a pharmaceutical drug on their exterior or in their mass that can be directed to the target cell and issued there. The size, charge, and chemical nature of NPs are critical and have a significant impact on both blood flow period and particle bioavailability within the body [172]. Numerous drug delivery approaches were developed to improve bioavailability, such as silica NPs [173], gold nanoparticles [116], phospholipid micelles [174], and PLGA/PLA NPs [175].

The NPs or drug compound are complexed with a particular bioactive component that unites a targeted site. Conjugates of gold NPs with antibiotics give encouraging outcomes in treating intracellular infections [176]. Active and passive targeting are two types of targeting. The accumulation of NPs or pharmaceutical materials at a particular site by physiochemical, extravasation, or pharmacological factors is referred to as passive targeting [177]. Methotrexate, for example, is a folic acid alternative that can disrupt cell folate metabolic activity and has been used as a cytotoxic anticancer drug. Perfect ferromagnetic granules—such as proteins, glycol-proteins, and other ligands—were used to enable adhesion while conserving bioactivity, AuNPs were also coated with poly (ethylene glycol), amino, or carboxyl groups. Ferromagnetic granules have also been used in vivo for a variety of applications including blood flow tracing, radionuclide angiography, and inducing clotting in arteriovenous malformations. Processes for making drug-loading NPs—such as post-loading, co-loading, and pre-loading—have been established. The biological importance of nanoparticles in drug loading is summarized in Table 1.

### 6.4. Immunoassay

Immunoassay is a useful methodology for diagnostic techniques and environmental control that relies on the communication of an antibody and a complement antigen. This is categorized as radioimmunoassay [178], fluorescent dye immunoassay [179], chemiluminescence immunoassay [180], and enzyme-linked immunosorbent assay predicated on protein labeling components (ELISA). NPs functionalized with antibodies, such as human IgG and antibodies against harmful bacteria, were used to develop various immunoassays [181]. Rather than classical monoclonal or polyclonal antibodies, immunosensors were created utilizing signal peptide shard variable recombinant antibodies (scFv). High contrast, controllable dimensions, frame and surface characteristics, ease of integrating various functionalities, and lengthy circulation period are some of the advantages of NPs [182]. Numerous NP devices are currently being developed for variety of clinical signs, such as superparamagnetic agents, metal NPs, liposomes, and others. The bioavailability, pharmacokinetics, toxicological, immunogenicity, and selectivity of every device differ. The innovation of immune and aptamer-based nanowire biosensors to recognize cancer biomarkers such as VEGF [183], CA125 [184], or SARS virus N-protein [185] showed tremendous sensitivity, suggesting that these nanodevices could be used for point-of-care clinical purposes. To acknowledge diverse diseases such as cancer, a multimode strategy is essential to improve efficacy of biosensors treatment. Zheng et al. [186] characterized nanowire clusters for multiplexed identification of many proteomic biomarkers at the pg/mL level, such as prostate-specific antigen (PSA), PSA-alpha1-antichymotrypsin, carcinoembryonic antigen, and mucin-1. Due to their controlled size and good biocompatibility with antibodies, proteins, RNA, and DNA, metallic NPs (Au NPs and Ag NPs) have been successfully used to label technology [187]. A immunogold silver staining (IGSS) tactic has been used to constrain antigens in cells or tissues, and it was recently discovered that strategy could boost the sensitivity of DNA array biomarkers [188]. Thus far, no attempts have been made to use the IGSS method in conjunction with 4 nm Pt NPs to improve efficacy and sensitivity of immunoassays. Monoclonal antibody immunoassays are presently used in research and diagnostics. Monoclonal antibodies are widely used in the innovation of sandwich assay methods [189].

In addition to colorimetric assays centered on AuNP immobilization or accumulation for in vitro diagnostic immunoassays, AuNPs and other plasmonic NPs could be used in lateral-flow. Pregnancy tests predicated on urine for protein markers, such as human chorionic gonadotropin (hCG) recognition, are identified diagnostic immunoassays [190]. Together, such immunoassay implementations to underline texture of NPs highlight biological authenticity of NPs in biological media and postulate cellular receptor involvement, and thus potential NP biological influence in cell systems [191]. Once administered openly, a few immunotherapeutics, such as proteins, have limited value delivery. As a consequence, NPs can significantly improve delivery by defending immunotherapeutics and trying to improve their communication with immune cells. The biological importance of nanoparticles based on immunoassay are summarized in Table 1.

## 7. Concluding Remarks and Future Perspectives

Certainly, nanotechnology is so far showing minor contributions toward the movement to individualized biomedicine and agriculture; nevertheless, the possibility of nanomedicine remains incontrovertible. Advances in science contribute in substances with distinct and significant structural, imaging, and catalytic functions. MNPs were examined extensively in terms of characteristics, synthesis methodologies, and surface functionalization for biomedical application efficiency in vivo and in vitro in this review. MNPs could be synthesized efficiently for biomedical purposes using a variety of methods. The stability, biocompatibility, and even solubility of IONPs can all be improved with surface coating, considerably expanding the range of applications for IONPs. Various synthesis approaches, reaction processes, performance, improvement, and prospective applications were explored as well. For the functionalization of MNPs, various chemical modalities (both synthetic and natural) have been investigated and found to be effective. The most important and related biomedical applications for NPs may be directed to the healthcare of aged individuals, most particularly with musculoskeletal system diseases. The development of improved bio-interfacial coating practices capable of imparting magnetic dispersion with long retention time in blood and functioning to engage in biorecognition binding events is a big challenge in the clinical use of NPs. Bonding of particular antibodies to NPs may enable particles to influence cells, like macrophages, which have receptors expressed on surfaces. Inflammatory response can be reduced through magnetic properties to focus macrophages or, if necessary, redirecting them from tissue. Improving achievement rate is critical for classification and much more cell managing. On the other hand, microfabrication provides plans that convey various mechanical, electrical, optical, and magnetic signals. These parts can be controlled in a variety of shapes and sizes, and their signals can be transferred with great accuracy.

Natural nanomaterials (NMs) have existed for a long time in the environment, and they have some processes in them to make living creatures safer. However, advances in the study of NPs have demonstrated that nanosized particles have some acute harmful effects in biological systems. Many countries have imposed restrictions and laws to reduce or eliminate the possible risks of engineered NMs in consumer products. In recent years, toxicity assessment of NMs has become a popular study concern all around the world.

The main areas expected to involve several applications include bioimaging, biomedical diagnostics, drug delivery, and tissue engineering studies. Research including stem cell differentiation and transplantation, as well as targeted drug delivery with real-time monitoring abilities will provide sought-after evidence as well. Soon, nanoparticulate drug loading systems can exploit many biological drugs with poor aqueous solubility, permeability, and less bioavailability. By preserving their stability and structure, NPs can alleviate some of these drugs’ unique problems. Furthermore, NPs provide a more advanced treatment by allowing for targeted loading and controlled release in clinical and agricultural trials.

## Figures and Tables

**Table 1:** Different NPs investigated for their precision use in biological sciences.

Nanoparticle	Bio-Application	Reference
Cadmium sulfide	Antimicrobial	[65]
Titanium oxide	Treatment of wound infection	[66]
Silver	Cytotoxicity	[67]
Zinc	Drug loading	[68]
Gold	Capping agent and medical field	[69]
Gold	Anticancer	[70]
Platinum	Antimicrobial and anticancer	[71]
Platinum	Cytotoxicity	[72]
Zinc	MRI and CT bioimaging	[73]
Titanium oxide	Drug delivery	[74]
Iron oxide	Drug carriers for cancer therapy	[75]
Gold	Ultrasound	[76]
Chitosan-gold	Synergistic gene/photothermal therapy	[77]
Gold	X-ray/CT scan	[78]
Platinum	Anti-fungal activity against *A. parasiticus* and *A. flavus*	[79]
Silver	Antiviral and antibacterial	[80]
Selenium	Biomedical applications	[81]
Silver	Showed antileishmanial effect in vivo	[82]
Iron oxide	Bactericidal against *Streptococcus*	[83]
Silver	Treat cancer, diabetes, and microbial infections	[84]
Silver	Antifungal	[85]
Zinc oxide	Antimicrobial activity	[86]
Silica	Nanocarriers for drug and gene delivery	[87]
Silver	Activity against *Pseudomonas aeruginosa*	[88]
Silica	In vitro and in vivo anticancer activity	[89]
Zinc	Drug delivery	[90]
Platinum	Induce apoptosis	[91]
Iron oxide	MRI imaging	[92]
Silicon	Ratiometric fluorescence Immunoassay	[93]
Gold	Mosquitocidal	[94]
Platinum	Environmental, biological, and catalytic applications	[95]
Iron oxide	Inhibition of MCF-7 breast cancer cells	[96]
Palladium	Biomedical applications	[97]
Silver	Biolarvicidal	[98]
Iron	Drug loading	[48]
Silver and Gold	Biomedical applications	[99]
Iron oxide	Biological applications	[100]
Silica oxide	Production of thermal and electric insulators gene delivery, drug carriers	[101]
Iron oxide	Antioxidant and antimicrobial	[102]
Silver oxide	Drug delivery, gene therapies, imaging	[103]
Silver and Gold	Antimicrobial	[104]
Silica	Antimicrobial	[105]
Platinum	Anticancer	[106]
Silver	Antifungal	[107]
Iron oxide	Drug delivery	[108]
Zinc oxide	Biomedical applications	[109]
Iron oxide	Drug-carrying vehicles	[110]
Silica	Drug loading	[111]
Silica	Activity as an antibiotic against intestinal bacterial infection	[112]
Silver	Antimicrobial	[113]

## Data Availability

No new data were created or analyzed in this study. Data sharing is not applicable to this article.
